# Assessing Violence Risk in Adolescents in the Pediatric Emergency Department: Systematic Review and Clinical Guidance

**DOI:** 10.5811/westjem.2021.1.49233

**Published:** 2021-05-19

**Authors:** Megan M. Mroczkowski, John T. Walkup, Paul S. Appelbaum

**Affiliations:** * Columbia University Irving Medical Center, Department of Psychiatry, New York, New York; † Ann & Robert H. Lurie Children’s Hospital of Northwestern University, Department of Psychiatry and Behavioral Sciences, Chicago, Illinois

## Abstract

**Introduction:**

Violence risk assessment is one of the most frequent reasons for child and adolescent psychiatry consultation with adolescents in the pediatric emergency department (ED). Here we provide a systematic review of risk factors for violence in adolescents using the risk factor categories from the MacArthur Violence Risk Assessment study. Further, we provide clinical guidance for assessing adolescent violence risk in the pediatric ED.

**Methods:**

For this systematic review, we used the preferred reporting items for systematic reviews and meta-analyses (PRISMA) 2009 checklist. We searched PubMed and PsycINFO databases (1966–July 1, 2020) for studies that reported risk factors for violence in adolescents.

**Results:**

Risk factors for adolescent violence can be organized by MacArthur risk factor categories. Personal characteristics include male gender, younger age, no religious affiliation, lower IQ, and Black, Hispanic, or multiracial race. Historical characteristics include a younger age at first offense, higher number of previous criminal offenses, criminal history in one parent, physical abuse, experiencing poor child-rearing, and low parental education level. Among contextual characteristics, high peer delinquency or violent peer-group membership, low grade point average and poor academic performance, low connectedness to school, truancy, and school failure, along with victimization, are risk factors. Also, firearm access is a risk factor for violence in children and adolescents. Clinical characteristics include substance use, depressive mood, attention deficit hyperactivity disorder, antisocial traits, callous/unemotional traits, grandiosity, and justification of violence.

**Conclusion:**

Using MacArthur risk factor categories as organizing principles, this systematic review recommends the Structured Assessment of Violence Risk in Youth (SAVRY) risk- assessment tool for assessing adolescent violence risk in the pediatric ED.

## BACKGROUND

Violence or aggression among adolescents is a common problem of enormous public health significance. Physical fighting is the most common form of violence in adolescents.^1^ In addition to the increased risk for injury and substance abuse, those who fight report less satisfaction with life, poorer relations with family and peers, and a worse perception of school. Within the past 12 months, 32.8% of high school-aged youth have been in a fight and 16.6% carried weapons to school.^2^ Since the 1980s, youths aged 10–17 years constituted less than 12% of the US population but have been offenders in 25% of serious violent victimizations.^3^

The evolution of violence can be conceptualized to begin in young childhood. Children first learn to manage aggression from their parents as toddlers; poor parenting, such as abuse, neglect, coercive parenting styles, antisocial modeling, and poor limit setting, may lead to an increased risk for violence.^4^ About 30% of those with oppositional defiant disorder go on to develop conduct disorder.^5^ Of those with conduct disorder, about 40% will progress to antisocial personality disorder.^6^

There are two main patterns of development of violence: early onset and late onset.^7^ Early-onset violence begins before puberty, accounts for 30% (+/− 15%) of serious violent offenders,—13% of whom go on to violent careers longer than two years—and is strongly associated with general offenses and substance use.^7^ In contrast, late-onset violence begins after puberty and accounts for 70% (+/− 15%) of serious violent offenders, 2% of whom go on to violent careers longer than two years.^7^ Late-onset violence is associated with weak social ties, antisocial and delinquent peers, and gang membership. 7

There are key differences between violent behavior in adolescents and adults.^7^ These differences can be categorized into epidemiology, diagnoses, behavior patterns, treatment, and legal status. In adolescents, compared to adults, violence is much more common and accounts for a higher proportion of all deaths, and violent careers are shorter; the first episode of serious violence most often occurs in adolescence, sometimes childhood, and rarely in adulthood.^7^ Psychotic disorder is much less common in adolescents who are violent than in adults. Adolescent violent behaviors tend to occur more in groups than adult violent behavior.^7^

Programs at all levels of schooling are effective in preventing violence. In addition to reducing aggressive and violent behaviors, these programs also improve school achievement and activity levels, and reduce truancy.^8^ In middle school, programs focus on disruptive behaviors, bullying, and general violence, while high school programs focus on violence, dating violence, and bullying. The programs that decreased violence most drastically were those taught by peers.^8^ Treatment for adolescents who are violent should consider both peer and family involvement.^7^ Adolescent legal status allows for legal consent for treatment to be provided by a legal guardian and, with some variation by age across states, hospitalization can occur over the patient’s objection with a legal guardian’s consent.^7^

Aggression and violence are one of the most frequent reasons for child psychiatry consultation on adolescents in the emergency department (ED).^9^ Assessment of violence risk may be required to determine appropriate disposition and avoid liability for untoward outcomes. Therefore, predicting who may become violent is of utmost importance. Unfortunately, predicting violence can be difficult; studies have shown that psychiatrist and nurse predictions of violence in both inpatient and community samples are poor, at times not differing from chance. 10

Assessing violence risk falls into the purview of pediatricians and child and adolescent mental health professionals. Following work in adult, actuarial risk-assessment scales, there has been progress in applying scales to adolescents.^11^ The two scales that have the strongest psychometric support are the Structured Assessment of Violence Risk in Youth (SAVRY) and the Psychopathy Checklist-Youth Version (PCL-YV). 12,13 However, neither these nor other scales are routinely used in clinical practice.

To equip both ED pediatricians and child and adolescent mental health professionals with the best knowledge to confront the assessment and treatment of aggression, we report a systematic review of the literature on risk factors for violence in adolescents in the community and characterize what is currently known using the risk factor categories from the MacArthur Violence Risk Assessment study as organizing principles; identify gaps in knowledge; and discuss recommendations for further research.^14^ We conclude with recommendations for assessing adolescent violence risk in the pediatric ED.

## METHODS

### Protocol and Registration

For this systematic review, we used the preferred reporting items for systematic reviews and meta-analyses (PRISMA) 2009 checklist. Full details of this review are listed below.

### Eligibility criteria and Data Sources

We searched *PubMed* and *PsycINFO* databases (1966–July 1,2020) for studies that reported risk factors for violence in adolescents. We also searched reference lists from identified reports for additional sources. We considered only articles published in English.

### Search

To create a comprehensive list of studies examining risk factors for adolescent violence, we used combinations of the following search terms (Figure).

#### PubMed database

risk factors AND violence AND juveniles (#66); risk factors AND violence AND juveniles AND review (#13); predictors AND violence AND juveniles (#8); predictors AND aggression AND juveniles (#5); predictors AND violence AND adolescents (#1107); risk factors AND violence AND adolescents (#7270).

#### PsycINFO database

risk factors AND violence AND juveniles (#63), risk factors AND violence AND juveniles AND review (#13), predictors AND violence AND juveniles (#17), predictors AND aggression AND juveniles (#10), predictors AND violence AND adolescents (#297); risk factors AND violence AND adolescents (#803).

### Study Selection

We included a study in our dataset if it examined or included risk factors for violence in adolescents. We defined adolescent as an individual between the ages of 11–18. Violence was defined as fighting, using a weapon in a fight, hitting or beating up someone, hurting someone badly enough to need bandages or a doctor, or using a weapon to obtain something. Violence did not include violence against oneself.

We excluded a study from the dataset if it had any of the following characteristics: 1) only included violence among inpatient populations; 2) focused solely on intimate partner violence; 3) was a review, letter or editorial; 4) had been withdrawn; or 5) only described clinical violence assessment practices of forensic evaluators. The lead investigator (MM) searched and vetted each prospective paper, sharing the descriptive information with co-authors (JW and PA) for their review and comments. The lead investigator, taking these comments, had the final say on study inclusion.

### Data Collection Process

We extracted data and recorded information on the details of where and how the study was conducted, sample characteristics, size of study, and how risk factors were measured.

### Data Items

We categorized the correlates of violence identified in the studies using the typology of the MacArthur risk assessment study: *Personal, Historical, Contextual and Clinical* characteristics.^14^ A risk factor was considered positive if there was a statistically significant (*P*<0.05) association with violence as an outcome. The number of total subjects in each row (N) in Tables 1–4 indicates the number of subjects in studies in which the results for that variable were significant.

### Risk of Bias in Individual Studies and Across Studies

We considered potential biases at the study level, broadly defined, focusing on flawed study design. Given that in this systematic review we considered studies with multiple outcome measures that differed across studies, standard metrics of bias in the literature (eg, publication bias) were inapplicable.

## RESULTS

### Study Characteristics

All but two of the studies in this review were surveys or longitudinal observational studies. There were no randomized controlled trials addressing violence risk in adolescents.

### Risk of Bias Within Studies

Many of the studies suffered from flaws in study design. Taken as a whole, the studies considered a constricted range of risk factors, weak criterion measures of violence, narrow study samples, and data gathered at a single site. These flaws are elaborated on in the Discussion section.

### Results of Individual Studies

*Personal characteristics* (Table 1) found to be correlates for violence in adolescents included male *gender*, *race* (Black, Hispanic, or multiracial), *religion* (no religious affiliation), *IQ* (lower IQ), and *age* (younger age). 15–28

*Historical characteristics* (Table 2) can be further organized within the following subcategories: *criminal history, disruptive behavior, parental criminal history, physical abuse*, and *family history*. Within the subcategory of *criminal history*, a younger age at first offense, higher number of previous criminal offenses, prior violence, and drug selling were found to be correlates for violence in children and adolescents. *Disruptive behavior* can be characterized by aggressiveness or fighting in childhood, cruelty to people, early antisocial influences or behaviors, conduct problems, under-controlled behavior at age six, carrying a weapon, and animal cruelty. *Parental criminal history* involves criminal history in either parent. *Physical abuse* is described as maltreatment starting in childhood or adolescence. *Family history* risk factors include the child’s parents experiencing poor child-rearing when they were children, low parental education level, and higher maternal antisocial personality disorder score, maternal bipolar disorder, interparental violence, family alcohol or drug use, and low parental support.^15^,^16^,^19^,^21^,^23^,^26^,^27^,^29^–^58^

*Contextual characteristics* (Table 3) found to be correlates for violence in adolescents include the categories of s*chool, social relations, firearm access, relationship with parents*, and *socioeconomic status*. Within the category of *school*, low connectedness or support at school, low grade point average, truancy, low school motivation, suspensions, feeling unsafe at school, poor study skills, school failure or repeating a grade, wanting to quit school, or feeling school discipline is unfair are all risk factors. *Social relations* that were risk factors included high peer delinquency, friends who use drugs, bullying others, victim of bullying, gang affiliation, sexually active, unsafe sex (in males), fewer friends committed to learning, dating violence, belonging to a sports team, peer pressure, and low peer support. *Firearm access* is a risk factor for violence in children and adolescents.^59^ Risk factors within the category *relationship with parents* include family strain, high parental stress, parental psychological aggression, parental non-authoritative behavior, poor relationship with parents, parent-child conflict, less parental control, rejecting parenting, and living in a single-parent household. *Socioeconomic status* risk factors include low socioeconomic status, exposure to community violence, drug use in the community, community disorganization, having five or more siblings, and living in a neighborhood where young people are in trouble.^17^–^19^,^22^,^24^,^27^,^30^–^32^,^35^–^37^,^45^–^47^,^49^–^52^,^55^,^57^,^59^–^80^

*Clinical characteristics* (Table 4) associated with correlates for violence in adolescents were organized into the following categories: *substance use; depressive symptoms; attention deficit hyperactivity disorder (ADHD); impulse control; temperament and personality trait;* and *psychopathy*. Cigarette, alcohol, and other illicit substances were found to be risk factors and can be classified under *substance use*. Symptoms related to *depression*, including suicide attempts, are risk factors for violence, as are *ADHD*, post-traumatic stress disorder, and *psychotic-like experiences*. *Impulse control* deficits, including lack of self-control, risk-taking behaviors, and previous unintentional injury, were also associated with violence risk. *Temperament and personality traits* that were risk factors include antisocial traits, callous/unemotional traits, grandiosity, justification of violence, intrapersonal strain, anger, perceived invulnerability to future events and the belief that damaging another’s property while intoxicated was acceptable, Cluster A and B personality traits, emotional distress, higher levels of aggressive beliefs, poor emotion regulation, and reduced likelihood of suppressing anger were also risk factors for violence.^15^,^16^,^19^–^22^,^24^,^27^,^30^,^32^,^35^–^37^,^42^,^45^,^47^,^49^,^51^,^52^,^56^,^57^,^60^,^62^–^66^,^68^,^69^,^71^,^75^,^81^–^90^

## DISCUSSION

### Summary of Evidence

From the studies included in our dataset, several risk factors were found in multiple studies and stand out clearly. Personal risk factors include male gender and race (Black, Hispanic or multi-racial), along with lower IQ and younger age. Historical risk factors include childhood aggressiveness in boys, childhood fighting, early antisocial influences, hyperactivity and withdrawal in childhood, child maltreatment, and higher maternal antisocial personality disorder score. Younger age at first offense and prior violence were described in a multitude of studies. These risk factors fit with the adage that “the best predictor of future behavior is past behavior,” in that those children who were aggressive or in fights were at risk for future violent behavior. Moreover, early influences are also apparent within this category; specifically, maltreatment as a child or early antisocial influences, especially by the mother, were risk factors. Children learn from the actions of their early caretakers, even if these are antisocial in nature. Additionally, children and adolescents who were themselves maltreated are at risk for perpetrating violence on others.

### Limitations of the Literature

The flaws identified in this body of research can be organized and addressed using the critique of violence research on persons with mental illness offered by Monahan and Steadman.^10^ They identified four problems: constricted range of risk factors; weak criterion measures of violence; narrow study sample; and data gathered at a single site.

#### Restricted range of risk factors

The first problem is that different studies focus on different risk factors, with no study looking comprehensively at the full range of risk factors. While studies may have included several risk factors, unless they are all measured simultaneously, it is unclear how they interact or whether one fully accounts for the variance that would otherwise be associated with the other. This limits the utility of the data for clinicians, who may be uncertain how much weight to give one or another variable in assessing violence risk.

Risk factors in studies of adolescents have focused on past history and symptom rating scales, such as the Brief Psychiatric Rating Scale. These variables are too narrow and may miss many key risk factors. For instance, risk factors should be studied in multiple domains, including historical and contextual, along with those within a single domain that may be theoretically related, such as impulsivity and anger management. In this review, studies did look at childhood traits such as hyperactivity, conduct problems, and aggressiveness, which may be a good start. Further, various symptoms have been studied, including depressive symptoms and substance abuse. However, it would be more meaningful to document changes in symptoms over time and explore how specific symptom clusters within a broader diagnosis may affect risk. Situational risk factors have been addressed, such as poor academics, truancy, peer delinquency, access to firearms, parental stress and low socioeconomic status, but not consistently across studies.

#### Weak criterion for violence

The second problem is weak criterion measures for violence. Typically, violence was defined in an undifferentiated manner, ie, all violent outcomes were treated the same. It may be helpful for researchers to define subtypes of violence, as predictors for one type of violence (eg, impulsive violence) may vary from another type (eg, gang violence). However, studies in our review rarely divided violent outcome by subtypes.

#### Narrow study samples

The third problem identified was narrow study samples. A majority of the studies in this review focused on populations of juvenile delinquents, schools in high-crime areas with low socioeconomic status, mental health clinics, and so-called at-risk youth. Broader samples of subjects should be sought. For example, studies should include both genders, those with and without a history of violence, and multiple socioeconomic statuses. Crucial for further research is the need to widen the inclusion criteria such that risk factors can be understood more universally.

#### Data gathered from single site

The fourth problem found was data gathered at a single site. When only one site is used, idiosyncratic aspects of the sample available, treatments used, and approaches to rating study variables can limit the generalizability of the data. Studies with larger samples and, therefore, more stable findings usually require research efforts to be coordinated across multiple sites. A few of the studies in this review were national in scope, in the United States and Finland, but the majority were limited to one or a small number of sites. As the research currently stands, groups have created their own lists of predictors and variables, which has led to disjointed findings in the literature. Ideally, groups of researchers should combine efforts in a multidisciplinary and multisite fashion to create common predictors and variables to study risk factors in large number of adolescents.

### Limitations of the Review

We did not rate the potential bias in individual studies. There were no randomized controlled trials identified in this search. A majority of the studies were surveys or longitudinal observational studies and, therefore, we did not include the study grade in our tables. Furthermore, we included only English-language papers, searching *PubMed* and *PsycINFO*, which may have led to the exclusion of some studies.

### Implications for Clinical Risk Assessment

Clinically, organizing risk factors by MacArthur risk factor categories may be useful as a means to carry out a risk assessment with an adolescent presenting to the ED with violence risk. Risk assessment may include interviews with the subject, caretaker, family member, and teacher, along with reviewing mental health, school and police records.^91^ Given the large number of variables that have been associated with violence and likelihood of significant overlap in the variance for which they account, risk assessment tools may be useful, as may tests of psychopathology, intelligence, and psychopathy. In a study of forensic evaluators, the most used of such tests were the Wechsler Intelligence Scales (75%), the Minnesota Multiphasic Personality Inventory (66.2%), and the SAVRY risk-assessment tool (35.1%). 91 Additionally, one third of clinicians surveyed always or almost always used the Psychopathy Checklist: Youth Version (PCL:YV).^91^ Each of these tests provides further information for risk assessment and includes a portion of the factors identified in this review.

The SAVRY is the violence risk-assessment instrument for adolescents most commonly used by forensic evaluators.^91^ Its rating form is organized into historical risk factors, social/contextual risk factors, individual/clinical risk factors, and protective factors.^12^ Historical risk factors include history of violence; early initiation of violence and exposure to violence at home; childhood history of maltreatment; parental/caregiver criminality; and poor school achievement. Social/contextual risk factors include peer delinquency; peer rejection; stress and poor coping; and poor parental management, among others. Individual/clinical risk factors include risk taking/impulsivity; substance use difficulties; anger management problems; attention deficit/hyperactivity difficulties; and low interest/commitment to school, among others. Protective factors include prosocial involvement; strong social support; strong commitment to school; and positive attitude toward intervention and authority.^12^

### Conclusions and Recommendations for Assessing Violence Risk in the Pediatric Emergency Department

Violence in adolescents is a problem with large public health significance. Its risk factors can be organized using the MacArthur risk assessment study categories. The Structured Assessment of Violence Risk in Youth is the most commonly used violence risk-assessment instrument for adolescents by forensic evaluators.^91^ Given this systematic review, we recommend its use in the pediatric ED to assess adolescent violence risk. Its rating form is organized into historical risk factors, social/contextual risk factors, individual/clinical risk factors, and protective factors.^10^ Overall, the SAVRY provides a comprehensive means of assessing risk factors as the literature now stands, and likely is best used in combination with clinical interviews and other testing.

## Figures and Tables

**Figure 1:**
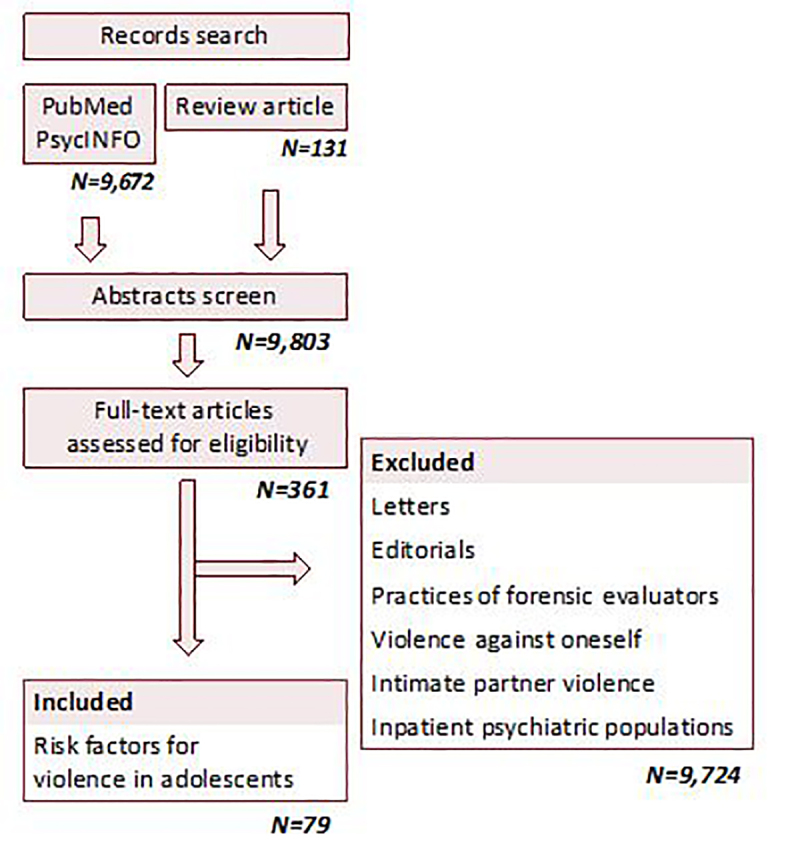
Search terms.

**Table 1:** Personal risk factors found to be correlates for violence in adolescents.

Risk factor	N (total)	References
Gender		
Male gender	33,902	15,16–19,20–24
Religion		
No religious affiliation	3,872	20
Race		
Black	3,107	16,22
Hispanic	84,734	25
Multiracial	2,305	28
IQ		
Lower IQ	588	26,27
Age		
Younger age	2,385	19

*IQ*, intelligence quotient.

**Table 2:** Historical risk factors.

Risk factor	N (total)	References
Criminal history		
Younger age at first offense	11,008	15,29–33
Prior violence	24,784	55–57,21,47,58
Drug selling	4,586	21
Arrests	3,818	55
Disruptive behavior		
Cruel to people	1,517	30
Childhood aggressiveness (boys)	415	54
Children characterized as under-controlled at age 6	731	53
Childhood fighting	808	16
Early antisocial influences	808	16
Conduct problems	11,580	27,36,50–52
Carrying weapon	29,520	47,49
Animal cruelty	542	23
Parental criminal history		
Parental or familial criminality	8,012	29,27
Physical abuse		
Physical abuse	172,957	38,40–48
Sexual abuse	140,021	38,39
Neglect	1,037	39
Witnessing abuse	136,549	38
Family history		
Poor child-rearing of parent	411	27
Low parental education level	5,385	35–37
Parental job loss	4,586	21
Higher maternal antisocial personality disorder score	2,562	19,26
Maternal bipolar disorder and perpetrating intraparental violence	120	34
Family alcohol or drug use	139,386	38,71
Low parental support	29,565	20,21,61
Parent convicted of crime	411	27

**Table 3:** Contextual risk factors.

Risk factor	N (total)	References
School		
Low connectedness/support at school	23,886	32,60,62
Low GPA	18,613	27,46,50,60,63, 64
Truancy	14,627	30,47
Low school motivation	1,517	30
Suspensions	12,703	55,63
Feel unsafe to go to school	46,756	49,65
Poor study skills	4,432	66
School failure/repeat grade	27,302	27,47,67
Wanting to quit school	3,955	51
Felt school discipline unfair	282	62
Social relations		
High peer delinquency	29,902	30,31,55,57,68–70,18,19,31,64, 66,71,72
Friends who use drugs	3,174	31,71
Bullying others	20,054	36,73,74
Victim of bullying or violence	21,789	24,71,75
Gang affiliation	1,642	46
Sexually active	2,299	22
Fewer friends committed to learning	2,055	31
Dating violence	1,080	31
Belonging to a sports team	1,642	46
Low peer support/peer rejection	28,898	61,70,72
Practicing unsafe sex (males only)	7,548	45
Peer pressure	4,056	70
Access to firearms	12,734	59,76
Relationship with parents		
Family strain	848	75
Parental psychological aggression	302	68
High parental stress	1,517	30
Parental non-authoritative behavior	2,335	35
Poor relationship with parents	9,603	31,45
Parent-child conflict	12,417	32,55,70,72
Less family involvement	1,080	31
Less parental control	1,080	31
Living in single-parent household	10,261	36,45
Rejecting parenting	310	52
Socioeconomic status		
Low socioeconomic status	49,113	27,30,61,77
Exposure to community violence	3,176	17,18,31,76,78–80
Drug use in neighborhood	4,626	55,64
Community disorganization	3,818	55
5+ siblings	511	27
Neighborhoods where young people were in trouble	808	32

*GPA*, grade point average.

**Table 4:** Clinical risk factors.

Risk factor	N (total)	References
Substance use		
Alcohol use	75,287	20,22,24,35,37,42, 47,49,63,66,81,82
Illicit drug use	121,891	56,63,69,83–85,19, 21,22,24,65,71,84, 86
Cigarette smoking	11,694	20,37,86
Depression		
Depression symptoms	4,491	30,35,37,68
Suicide attempt	16,410	49
PTSD	3	90
ADHD	10,209	16,27,32,36,60,64, 66
Psychosis-like experiences	18,104	24
Impulse control		
Lack of self-control	1,100	15,87
Risk-taking behaviors	9,770	27,45,57,75
Previous unintentional injury	337	37
Temperament and personality traits		
Antisocial traits or favorable attitude toward antisocial behavior	7,989	19,51,56,57,68,71
Grandiosity	974	89
Justification of violence	974	89
Anger	5,312	20,69
Callous/unemotional traits	3,019	36,56,69
Perceived invulnerability to future events	2,335	35
Belief that hurting another’s property while intoxicated is acceptable	1,332	84
Cluster A and B personality traits	717	88
Emotional distress	1,719	87
Poor emotion regulation	310	52
Higher levels of aggressive beliefs	1,719	87
Less likely to suppress anger	282	62

*PTSD*, post-traumatic stress disorder; *ADHD*, attention deficit hyperactivity disorder.
